# Production and Characterization of Chimeric Monoclonal Antibodies against *Burkholderia pseudomallei* and *B. mallei* Using the DHFR Expression System

**DOI:** 10.1371/journal.pone.0019867

**Published:** 2011-05-09

**Authors:** Hyung-Yong Kim, Shien Tsai, Shyh-Ching Lo, Douglas J. Wear, Mina J. Izadjoo

**Affiliations:** 1 Department of Environmental and Infectious Disease Sciences, Armed Forces Institute of Pathology and American Registry of Pathology, Washington, D. C., United States of America; 2 Division of Cellular and Gene Therapies and Division of Human Tissues, Center for Biologics Evaluation and Research, U. S. Food and Drug Administration (FDA), Bethesda, Maryland, United States of America; Indian Institute of Science, India

## Abstract

*Burkholderia pseudomallei* (BP) and *B. mallei* (BM) are closely related gram-negative, facultative anaerobic bacteria which cause life-threatening melioidosis in human and glanders in horse, respectively. Our laboratory has previously generated and characterized more than 100 mouse monoclonal antibodies (MAbs) against BP and BM, according to in vitro and in vivo assay. In this study, 3 MAbs (BP7 10B11, BP7 2C6, and BP1 7F7) were selected to develop into chimeric mouse-human monoclonal antibodies (cMAbs) against BP and/or BM. For the stable production of cMAbs, we constructed 4 major different vector systems with a dihydrofolate reductase (DHFR) amplification marker, and optimized transfection/selection conditions in mammalian host cells with the single-gene and/or double-gene expression system. These 3 cMAbs were stably produced by the DHFR double mutant Chinese hamster ovarian (CHO)-DG44 cells. By ELISA and Western blot analysis using whole bacterial antigens treated by heat (65°C/90 min), sodium periodate, and proteinase K, the cMAb BP7 10B11 (cMAb CK1) reacted with glycoproteins (34, 38, 48 kDa in BP; 28, 38, 48 kDa in BM). The cMAb BP7 2C6 (cMAb CK2) recognized surface-capsule antigens with molecular sizes of 38 to 52 kDa, and 200 kDa in BM. The cMAb CK2 was weakly reactive to 14∼28, 200 kDa antigens in BP. The cMAb BP1 7F7 (cMAb CK3) reacted with lipopolysaccharides (38∼52 kDa in BP; 38∼60 kDa in *B. thailandensis*). Western blot results with the outer surface antigens of the 3 Burkholderia species were consistent with results with the whole Burkholderia cell antigens, suggesting that these immunodominant antigens reacting with the 3 cMAbs were primarily present on the outer surface of the *Burkholderia* species. These 3 cMAbs would be useful for analyzing the role of the major outer surface antigens in Burkholderia infection.

## Introduction

*Burkholderia pseudomallei* (BP), the causative agent of melioidosis, is a gram-negative, facultative anaerobic, motile bacillus commonly found in the soil and stagnant waters [1]. BP infection is often due to either direct inoculation into wounds and skin abrasions or inhalation of contaminated materials [2], [3]. The clinical manifestation ranges from subclinical to acute localized, acute septicemic and chronic forms [4]. Recently, BP has been recognized as a major cause of community-acquired septicemia, resulting in significant mortality [5]. Moreover, numerous studies revealed that BP could be intrinsically resistant to many antibiotics. Despite therapeutic regimens with certain antibiotics, the mortality rate of melioidosis remains very high [6]. *B. mallei* (BM), a host-adapted pathogen that does not normally persist in nature, causes glanders in horse. Some studies indicated that BM is highly infectious in humans by aerosol route [7]. Thus, there are true concerns that BP and BM may be used as biological warfare agents (BWA) [8]. No effective vaccines or therapeutics of either melioidosis or glanders currently exist.

The only countermeasure providing a state of “immediate immunity” against these biowarfare agents is neutralizing antibodies. Unlike vaccines, antibodies can confer passive protection regardless of the immune status of the infected host. In comparison with antimicrobial therapy, antibody therapy against many potential BWAs such as *Bacillus anthracis*, *Francisella tularensis*, and *Yersinia pestis* is significantly promising due to high specific function and low toxicity [9]. Currently, specific antibodies that protect against infections of highly pathogenic BP and BM that military or civilian populations may encounter in biological warfares have not been developed. Basic Local Alignment Search Tool (BLAST) comparisons of the genomes indicated that the genes conserved between BP and BM are 99% identical at the nucleotide level [10], [11]. The extremely high homology among BP, BM, and *B. thailandensis* (BT) would allow for only small window of antigenic difference among these species of the Burkholderia bacteria. The main antigenic differences between BP and BM appeared to reside only in the O-capsular polysaccharides (PS) moiety of their lipopolysaccharides (LPS) structure. However, some BM strains might lack the O-PS moiety in their LPS structure. On the otherhand, different strains of BP were found to posses LPS with different chemical structure of the O-PS (O-PS I and O-PS II) [12]. Serological studies also revealed BP and BM are antigenically closely related [13]. Thus, it would be extremely difficult to obtain a single MAb that can both recognize all different clinical isolates of BP and at the same time differentiate them from those of BM as well as BT. Development of MAbs that can differentiate between all strains of BP and BM from other non-pathogenic *Burkholderia* species has been very challenging due to the close homology. However, if the MAbs developed were to be used for therapeutic and not diagnostic purposes, MAbs that react strongly to both BP and BM are highly desirable. Furthermore, to design therapeutic antibodies for human diseases, it is important that the selected MAbs react not only to the particular strain of bacteria used as the immunogen, but to as many different strains and clinical isolates of these two closely related species of bacterial pathogens as possible [14], [15].

In our previous studies, total 108 mouse MAbs against BP and/or BM have been generated, characterized, and categorized into 8 groups (from A to H) on their binding patterns against a panel of 11 species of the *Burkholderia* bacteria and the reactive antigens [PS, LPS, and (glyco)proteins] recognized by each MAb [16], [17]. Most importantly, many of these MAbs showed good protective efficacy against both pathogenic *Burkholderia* bacteria by an *in vitro* opsonic assay using differentiated HL-60 cells as phagocytes and *in vivo* protective efficacy of selected MAbs against intranasal challenge of BP and BM in mice. When compared individually, both anti-PS and anti-LPS MAbs performed better in mouse protection than the anti-glycoprotein MAbs. Some of these MAbs could potentially be developed into useful therapeutics in treating the devastating diseases caused by BP and BM [18]. Other investigators also showed that the intranasal challenge with a sub-lethal dose of the bacteria has shown that MAbs against PS, LPS, and glycoproteins of BP and/or BM significantly reduce lethality of infections in mice. However, these MAbs fail to achieve full protection, especially with high dose challenges [19], [20].

Development of high-performance cMAbs which could be used as therapeutics in exposed individuals is urgently needed to fill the current gap in defense against BWAs. In this study, three major surface antigen (glycoprotein, PS, and LPS)-reactive MAbs (BP7 10B11, BP7 2C6, and BP1 7F7) against BP and/or BM were selected to further develop chimeric MAbs (cMAbs) for human therapeutics. The ultimate goal of this study is to develop superactive MAbs that can be used as therapeutics against BM and BP infections. Humanization of the target MAbs could minimize possible side effects when used as human therapeutics [21], [22]. Therefore, the chimerization of these 3 MAbs against BP and/or BM is an initial step in the development of MAb-based therapeutics.

The utilization of bicistronic retroviral expression vectors containing the gene of interest and an amplifiable marker gene has been shown as an effective method in obtaining stable cell lines that express high levels of the cMAb of interest [23]–[25]. However, experience with the use of such vectors in high level expression of cMAbs is currently limited. In this study, to obtain sufficient quantities of cMAbs, we established a series of technology to develop cell lines producing of neutralizing cMAbs against BP and/or BM. This include (i) construction of four major different mammalian expression vector systems with a dihydrofolate reductase (DHFR) amplification marker, (ii) optimization of transfection/selection conditions in Chinese hamster ovarian (CHO) cells and other common mammalian host cells with the single-gene (heavy or light chain) vectors and double-gene vector (both heavy and light chain in the vector) system, (iii) stable cMAb production by CHO-DG44 cells, and (iv) affinity purification. These techniques are useful for the development of stable cMAbs against targeted BWA for therapeutic purposes.

## Materials and Methods

### Construction of expression vectors with a DHFR amplifiable marker

**Figure 1:**
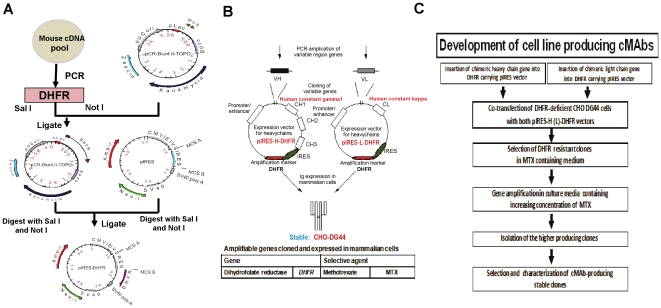
Expression system for production of stable cMAbs. (A) The pIRES-DHFR vector was constructed by cloning the modified DHFR gene into the MCS-B of the pIRES vector. The target chimeric H and L genes were separately cloned into the MCS-A of the pIRES-DHFR vector. (B) Only mouse variable genes (V_H_ and V_L_) of the target MAbs (BP7 10B11, BP7 2C6, and BP1 7F7) in each vector were replaced for stable production of cMAbs. (C) The flow chart shows the key procedures in the development of cMAb producing cells.

To obtain high level expression constructs we modified the pIRES (Clontech, Palo Alto, CA) vector containing two multiple cloning sites (MCS) A and B that allows expression of two genes of interest. Briefly, the PCR-amplified DHFR gene (564 bp) from mouse cDNA pools was synthesized by a specific primer set (forward primer with a *Sal* I site: 5′-GCTG CGTCGACCATCATGGTTCGACCATTGAACTGC-3′; reverse primer with a *Not* I site: 5′-GAACTTGAGCGGCCGCAAGCATCTTCTTGTTAGTC-3′). The DHFR gene (595 bp) was cloned into the PCR Blunt II-TOPO plasmid using the TA cloning kit (Invitrogen, Carlsbad, CA) and sequenced. To make the pIRES-DHFR vector system, the DHFR gene digested with *Sal* I and *Not* I was inserted into MCS-B site separated from MCS-A by an internal ribosome entry site (IRES) sequence (581 bp) in the pIRES vector (Fig. 1A).

To generate single-gene vector [pIRES-H(L)-DHFR] systems, mouse variable regions of the heavy and light chains were amplified by reverse-transcription PCR (RT-PCR). Total RNA was extracted from the 3 selected hybridoma clones (BP7 10B11, BP7 2C6, and BP1 7F7) (5×10^6^ cells each (Table 1) by TRIzol reagent (Invitrogen) according to manufacturer's instruction. For cDNA synthesis, total cellular RNA (2 µg) was reverse transcribed as previously described [26]. For PCR, the cDNA (2 µl) was amplified in a 50-µl reaction mixture containing 1× PCR buffer (10 mM Tris-HCl [pH 8.3], 50 mM KCl, 1.5 mM MgCl_2_), 0.2 mM dNTPs, and 0.4 µM (each) primer mixture combinations with homology to mouse heavy and light chain [For the V_H_ chain: one set of forward-reverse primer mixture (GE Healthcare, Piscataway, N.J.); For the V_L_ chain: 6 forward primer and 4 reverse primer mixtures (GE Healthcare)] in a DNA thermal cycler (BioRad, Hercules, CA). To reduce nonspecific priming, all PCRs were performed by the hot-start method as previously described [26]. To identify the consensus sequences of each variable gene, the target PCR products (V_H_: 351 bp; V_L_: 324 bp) were directly cloned into the PCR4 TOPO vector (Invitrogen) and their variable sequences were determined by multiple sequence alignments with mouse antibody sequence data. To introduce the chimeric heavy or light chains into the MCS-A in the pIRES-DHFR plasmid, the human constant regions of both chains (HcG1: 990 bp; HcK: 324 bp) were also amplified from human cDNA pools using the designed primer sets (HcG1-F: 5′-GCCTCCACCAAGGGCCCATCGG-3′ and HcG1-R: 5′-TTTfoACCCGGAGACA GGGAGAGGC-3′; HcK-F: 5′-CGAACTGTGGCTGCACCATCTG-3′ and HcK-R: 5′-CTAACACT CTCCCCTGTTGAAGC-3′). After the sequences of all PCR-amplified products were confirmed, the full-length chimeric heavy (light) chains were assembled by the overlapping PCR technique. A freshly prepared full-length chimeric heavy (light) chain was transformed into the DH5α competent cells (Invitrogen) and sequences of the plasmid DNAs prepared from more than 10 different clones were confirmed.

**Table 1:** Three selected hybridoma clones to convert stable cMAbs against BP and BM.

cMAb ID	3 selected hybridomas converted into cMAbs^a^				Chimeric chain ID^b^	
	Hybridoma clone ID	Antigen injected	Isotype	Cross-reactivity	Heavy(mVH+HcG1)	Light(mVL+HcK)
cMAb CK1	BP7 10B11	BP 430	IgG1/κ	BP/BM: +; BT: −	H1	L1
cMAb CK2	BP7 2C6	BP 430	IgG2a/κ	BP/BM: +; BT: −	H2	L2
cMAb CK3	BP1 7F7	BP 8324	IgG3/κ	BP/BT: +; BM: −	H3	L3

^*a*^These 3 hybridoma clones were selected by their binding specificities, antigen reactivities to their bacterial cell components, and in vitro/vivo neutralizing activities against *B. pseudomallei* (BP) 8324 and/or *B. mallei* (BM) ATCC 23344. BT: *B. thailandensis*.

^*b*^Each chimeric chain was assembled by the overlapping PCR. mVH: mouse variable heavy chain; HcG1: human constant gamma 1; mVL: mouse variable light chain; HcK: human constant kappa chain.

**Figure 2:**
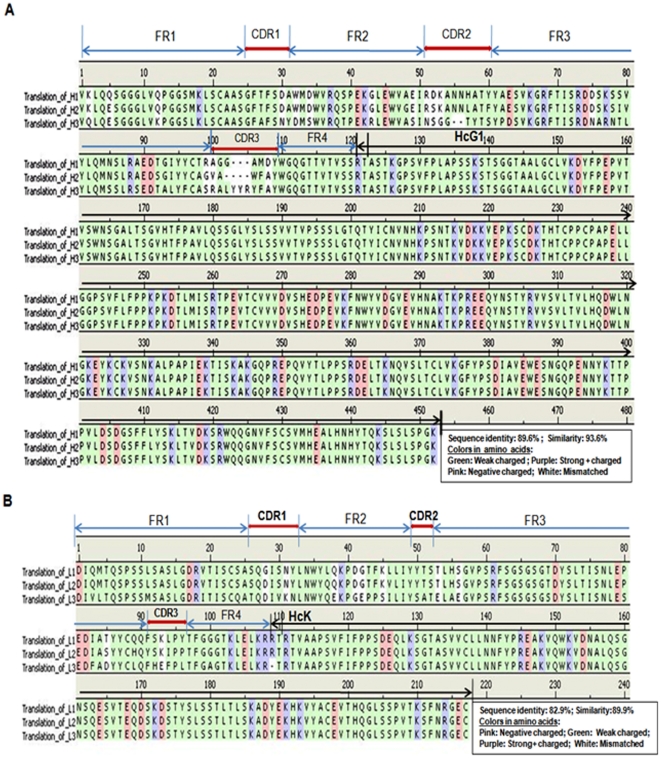
Multiple sequence alignment of three cMAbs CK1, CK2, and CK3. Sequences of each chimeric chain [(A) heavy chain: H1, H2, and H3; (B) light chain (L1, L2, and L3)] were ordered by FRs, CDRs, junction between mouse variable gene, and human constant genes (HcG1 and/or HcK) indicated on the basis of charge difference of amino acids in each chain. GeneBank access numbers: H1 (GU350787), H2 (GU350788), and H3 (GU350789); L1 (GU350790), L2 (GU350791), and L3 (GU350794).

To identify the hypervariable loop regions of each chimeric chain, BLAST search was performed against pre-compiled loop databases in *the Input Protein Molecule* (Discovery Studio 2.5; Accelrys, San Diego, CA). An alignment was created for each loop against its best hits from the BLAST searches. Multiple sequences of each chimeric chain were aligned by frame regions (FRs), complementarity-determining regions (CDRs), and human constant genes (HcG1 or HcK) using the ClustalW multiple sequence alignment software, Discovery Studio 2.5 (Accelrys) (Fig. 2).

**Figure 3:**
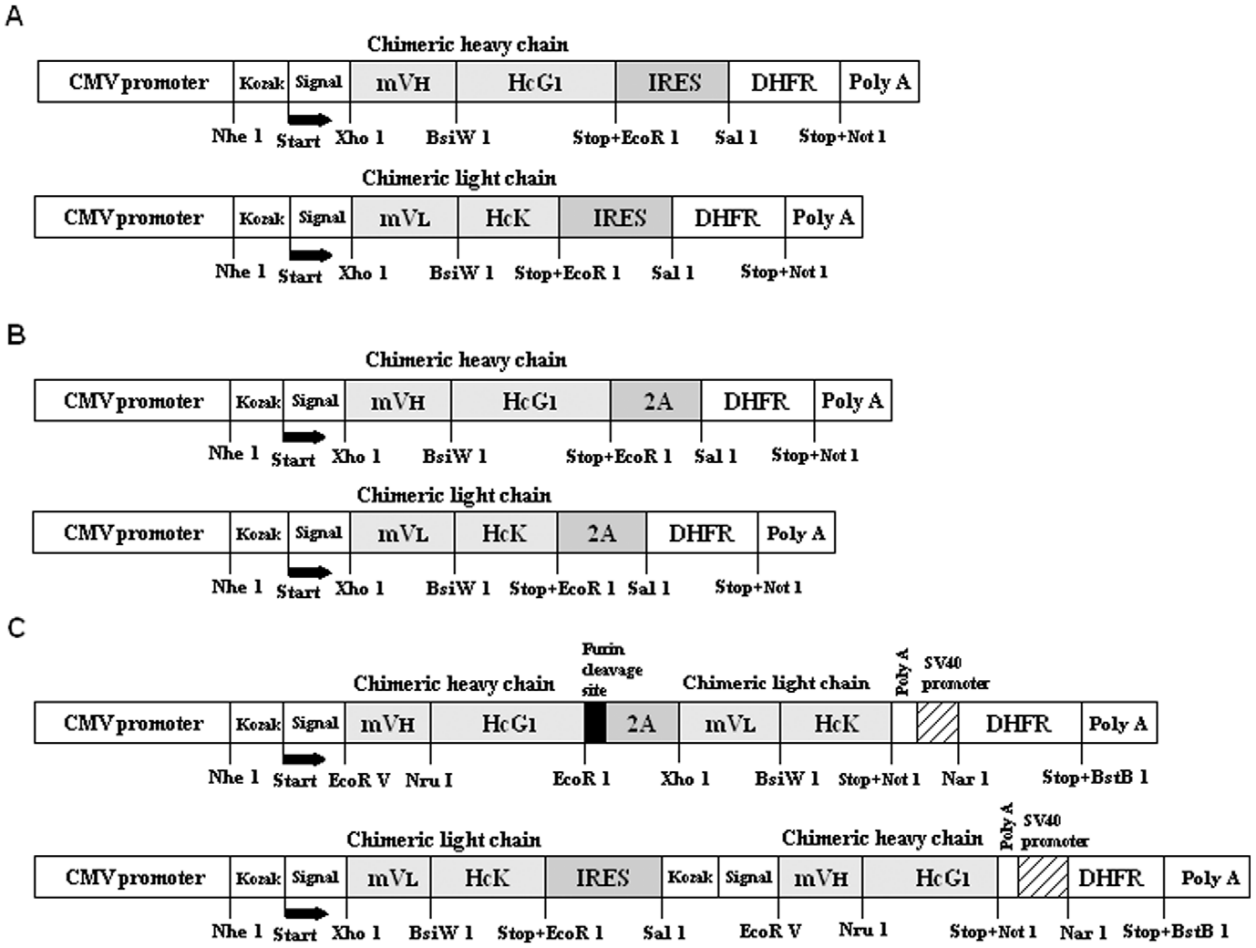
Chimeric antibody expression cassettes. (A) Single gene (chimeric heavy or light chain) was linked by IRES sequence in the bicistronic pIRES-DHFR vectors. (B) Single gene (chimeric heavy or light chain) was linked by FMDV-2A sequence in the bicistronic p2A-DHFR vectors. (C) Two genes (chimeric heavy and light chain) were linked by FMDV-2A or IRES sequence in the same bicistronic vector.

The full fragments of each chimeric chain contain restriction sites (*Nhe* I at 5′end and *EcoR* I at 3′end), Kozak sequence (CTCACCATG), synthetic leader sequences [GeneBank access numbers: H (GU350792); L (GU350793)] derived from consensus signal sequence of Ig genes, chimeric heavy (light) chain containing unique restriction sites between junctions [(*Xho* I-mV_H_-*BsiW* I-HcG1) or *Xho* I-mV_L_-*BsiW* I-HcK)], and stop codon (Fig. 3A). To construct the p2A-H(L)-DHFR vector system containing the foot and mouse disease virus (FMDV)-derived 2A self-processing sequence (APVKQTLNFDLLKLAGDVESNPGP), the cDNA oligo for 24-amino acid FMDV-2A peptide was synthesized (Sigma Life Science, Woodlands, TX). The short 2A DNA sequence (72 bp) was replaced with IRES sequence (581 bp) from the pIRES-H(L)-DHFR vectors to express cMAbs from a single open reading frame (Fig. 3B).

To generate the double-gene vector (pH-Fu2A-L-DHFR and pIRES-L-H-DHFR) systems, which contain both heavy and light chains in the vector, the DHFR gene at the MCS-B site in both plasmids (p2A-H-DHFR and pIRES-L-DHFR) was replaced with the neomycin structural gene in the neomycin cassette. To construct the pH-Fu2A-L-DHFR plasmid, the furin cleavage site sequence (RAKR) [27] was added by PCR between the chimeric heavy chain and the 2A sequence. The full-length cassette [(*Nhe* I-Kozak sequence-synthetic leader sequence for the heavy chain-chimeric heavy chain containing unique restriction sites between the junctions (*EcoR* V-mV_H_-*Nru* I-HcG1-*EcoR* I)-Furin cleavage site (RAKR)-2A sequence-chimeric light chain containing unique restriction sites between the junctions [(*Xho* I-mV_L_-*BsiW* i-HcK without Kozak and synthetic leader sequences)-stop codon-*Not* I)] was assembled by the overlapping PCR. After the sequence was confirmed, the full cassette was digested with *Nhe* I and *Not* I, cloned into the prepared p2A-H-DHFR plasmid (DHFR gene at neomycin cassette) (Fig. 3C, top construct). To make another double-gene vector system (pIRES-L-H-DHFR) containing IRES sequences between the junctions of light and heavy chains, after the pIRES-L-DHFR plasmid was double-digested with *Sal* I and *Not* I, the chimeric heavy chain containing unique restriction sites between junctions (*Sal* I-Kozak sequence-synthetic leader sequence-*EcoR* V-V_H_-*Nru* I-HcG1)-stop codon-*Not* I) was cloned into the downstream of IRES sequence (Fig. 3C, bottom construct).

### Optimization of transfection and selection

To determine the optimum plasmid DNA concentration and most favorable host cells for the transient expression of cMAbs using the pIRES-H(L) DHFR system, different amounts of the combined plasmid DNAs (5, 10, 20, and 40 µg) at different molar ratios (H∶L = 1∶1, 1∶2, 1∶4, 2∶1, 4∶1) in a 10-cm culture dish, and 8 common mammalian cells (CHO-K1, CHO-DG44, 293T, COS-7, BHK-21, SP2, P3×36, and IM-9) (Table 2) were used to optimize transfection conditions using either the lipofectamine (LF)-2000 or the LF-LTX reagent (Invitrogen). Two days prior to scheduled transfection, the cells were seeded at 15 ml in a 10-cm dish at a density of 5×10^5^ cell/ml in culture media containing 10% dFBS (Invitrogen). For each culture dish to be transfected, the plasmid DNAs (5, 10, 20, and 40 µg) at different molar ratios (H∶L = 1∶1, 1∶2, 1∶4, 2∶1, and 4∶1) were diluted into 800 µl of Opti-MEM I without serum. In a separate tube, 80 µl of LF-2000 or LF-LTX with 10 µl of PLUS reagent (Invitrogen) were diluted into 720 µl Opti-MEM I medium and incubated for 5 min at room temperature. The diluted DNAs were combined with the diluted LF-2000 or LF-LTX reagent, incubated at room temperature for 30 min to allow DNA-LF reagent complexes to form. After adding additional 6 ml Opti-MEM I medium to DNA-lipofectamine complexes (∼1,600 µl), the tubes were mixed gently, and plated onto the 10-cm culture dish prepared. The cells were incubated at 37°C in a CO_2_ incubator for 6 h. Six hours post-stimulation with the DNA-LF complex in the Opti-MEM I medium, the cells were washed once with PBS (137 mM NaCl, 10 mM Na_2_HPO_4_, 2.7 mM KCl, and 1.8 mM KH_2_PO_4_ [pH 7.2]), and changed with the regular culture medium containing 10% dFBS. The cells were incubated at 37°C in a CO_2_ incubator for additional 16 h to recover from any cytotoxicity from lipofectamine reagents or plasmid DNA itself freshly prepared from *E. coli*. After 16-h recovery with the regular medium supplemented with 10% dFBS, the media was replaced with the serum-free culture medium. After 72-h incubation, the culture supernatants were harvested, and the binding properties and cMAb levels were examined at each condition.

**Table 2:** Transient production of the 3 cMAbs by 8 common mammalian host cells^a^.

Cell line	Description	Concn^b^ (ng/ml)		
		CK1	CK2	CK3
CHO-K1	Chinese hamster ovarian cell - Used with transient expression and glutamine synthetase system	370±52	357±47	434±86
CHO-DG44	Chinese hamster ovarian cell - Used for DHFR coamplification to make stable clones for cMAbs	37±6	33±5	173±12
293T	Transformed human embryonic kidney cell with T antigen - Used for transient small-scale production	167±7	37±3	404±45
COS-7	Transformed African green monkey kidney cell -Used for transient small-scale production	226±10	196±8	441±72
BHK-21	Baby hamster kidney cell - Used for stable gene integration	35±7	38±6	177±13
Sp2/0-Ag14	Mouse myeloma cell - Used for obtaining hybridoma which produce MAbs as a fusion partner	<4	<4	<4
P3×36-Ag8.653	Mouse myeloma cell - Used for obtaining hybridoma which produce MAbs as a fusion partner	<4	<4	<4
IM-9^c^	EBV-transformed human B-lymphoblast cell – Derived from the blood of a patient with multiple myeloma	NT^d^	NT	424±57

^*a*^These cell lines were used to select a maximum producer by transient expression using the pIRES-H/L-DHFR vector system. These cells (∼10^6^ cells/10 cm dish) were incubated for 72 h after cotransfection with complex mixtures of each pIRES-H (L)-DHFR DNAs (10 µg/dish) and LF-2000.

^*b*^Culture supernatants of each cMAb were used to measure the cMAb levels by a modified human IgG1 ELISA. The assay detection limit of cMAb was 4 ng/ml. The values are the means ± standard deviations of 3 independent experiments.

^*c*^Since the transformed IM-9 cell line has a machinery to produce human IgG/κ chain, the antibody levels detected in the assay system were from the majority of endogenous human IgG/κ, not cMAbs.

^*d*^NT: Not tested.

### Stable expression of three cMAbs

To develop cell lines producing high levels of 3 cMAbs (CK1, CK2, and CK3), the DHFR gene-deficient CHO-DG44 cells obtained from Dr. Larry Chasin (Columbia University) were propagated in α-MEM with nucleosides and deoxynucleosides (Invitrogen) supplemented with 10% dFBS (Invitrogen). Two days before transfection schedule, the cells were trypsinized and seeded at 15 ml in a 10-cm culture dish at a density of 5×10^5^ cell/ml in α-MEM media containing 10% dFBS without antibiotics, so that they were 90–95% confluent on the day of transfection. For each culture dish to be transfected, the plasmid DNAs (10 µg each) [H∶L molar ratio = 1∶1 for pIRES-H(L)-DHFR] into 800 µl of Opti-MEM I without serum were diluted. In a separate tube, 80 µl of LF-LTX with 10 µl of PLUS reagent was diluted into 720 µl Opti-MEM I medium and incubated for 5 min at room temperature. The diluted DNAs were combined with diluted LF-LTX reagent and incubated at room temperature for 25 min to allow DNA-lipofectamine reagent complexes to form. After adding an additional 6 ml Opti-MEM I medium to DNA-lipofectamine complexes (1,610 µl), the dishes were mixed gently, and incubated at 37°C in a CO_2_ incubator for a total of 6 h. Twenty four hours post-transfection, the media was removed, washed once with PBS, and changed with the DHFR-selection media (α-MEM without nucleosides and deoxynucleosides) (Invitrogen) supplemented with 10% dFBS until they were ready to split. Stable CHO-DG44 cell clones producing cMAbs against BP or BM were identified by ELISA, isolated by using cloning cylinders, and amplified in culture media with increased levels of selective agent methotrexate (MTX, ICN, Costa Mesa, CA). The stable cell lines expressing high levels of antibodies were selected and re-tested for their binding affinity against BP. The selected stable cell clones were frozen for future large-scale production use.

### Production and purification

When stably transfected CHO-DG44 cell lines producing the selected cMAbs were established by bicistronic vectors containing the DHFR amplification system, the clones with the highest expression levels were grown in multiple flasks using serum-free CHO medium (Invitrogen) to obtain sufficient quantities for further use. Normally, without MTX amplification, when the transfected stable cell lines, secreting up to approximately 0.01 pg/cell/day were established, the cMAb-containing medium was harvested, centrifuged, and filtered (0.45 µm membrane).

To analyze cMAb production levels, the cMAb CK3 transfectoma CHO-DG44 cell clone was grown in batch culture to produce a sufficient amount of cMAb CK3. The supernatant was harvested after 10 days in batch culture by centrifugation at 300×g for 5 min. The cMAb CK3 supernatant samples were purified using an ÄKTAprime plus (GE Healthcare). The cMAb CK3 purification yield was investigated utilizing an affinity chromatography resin, a HiTrap rProtein A-Fast Flow column (GE Healthcare). In brief, samples were loaded and eluted at slow flow rates ranging from 0.25 to 0.5 ml/min. After direct loading of culture supernatant (∼1 L), the column was washed with 10 column volumes of equilibration buffer (20 mM sodium phosphate containing 150 mM NaCl, pH 7.5) and eluted in 100 mM glycine buffer (pH 3.0). The pH of the elution was immediately neutralized to pH 7.0 using 1 M Tris buffer (pH 10.0). The pooled cMAb CK3 fraction no. 1-4 (1 ml/tube) was concentrated and dialyzed in a Slide-A-Lyzer, MWCO 10K (Thermo Scientific). The cMAb CK3 concentration was measured by the modified human IgG1 ELISA, stored at −20°C for further use to examine any changes of their binding to specific Burkholderia bacterial antigens and their physical properties during humanization process from the original mouse MAb BP1 7F7.

### Expression analysis of stable cMAb CK3 by CHO-DG44 cells

To examine whether the DHFR gene in the bicistronic pIRES-H(L)-DHFR vector contributes the increase of cMAb expression, leading to the high-level production of stable cMAb against BP, total RNA was extracted from cMAb CK3 transfectoma CHO-DG44 cells (5×10^6^ each) cultured for 7 day at different MTX levels (0, 1, 4, 16, and 64 µM) by TRIzol reagent (Invitrogen). Total cellular RNA (2 µg) was reverse transcribed as described previously [26]. The cDNAs (2 µl each) were used to amplify H3, L3 chain, and DHFR gene including a house keeping hamster β-actin gene [28] (GeneBank access number: AJ312092) as a control by the PCR (50-µl reaction) using specific primer sets (H3-F: 5′-CGGCAGCCTCGAGGTGCAGCT GCAGGAGTCAGGGGG-3′ and H3-R: 5′-AAAGGGAAGAATTCGCGGCCGCTTATTTACCCGGA GACAGGG-3′; L3-F: 5′-GATGTCTCGAGGACATTGTGCTGACCCAGTC-3′ and L3-R: 5′-AAAG GGAAGCGGCCGCGAATTCCTAACACTCTCCCCTGTTGA-3′; DHFR-F: 5′-TCGACCATCATGG TTCGACC-3′ and DHFR-R: 5′-GCAAGCATCTTCCTGTTAGT-3′; β-actin-F: 5′-TCTACAACGA GCTGCG-3′ and β-actin-R: 5′-CAATTTCCCTCTCGGC-3′) to examine the effect of DHFR amplification on CK3 mRNA expression. PCR was conducted for 30 cycles in all experiments, except for β-actin mRNA (35 cycles). After PCR, 10 µl of each PCR product was electrophoresed in 1.5% agarose gel containing EtBr. DNA size markers (1 Kb Plus) were run in parallel. The cMAb CK3 production levels (7-day culture period) at different MTX concentration were measured by the modified human IgG1 ELISA.

### Treatment of bacteria cells

To analyze reactive antigens by three cMAbs, *B. pseudomallei* (BP) 8324, *B. mallei* (BM) [American Type Culture Collection (ATCC) 23343, Manassas, VA)], and *B. thailandensis* (ATCC 700388) were grown separately in Luria-Bertani (LB) medium at 37°C with shaking overnight. Bacterial cells were collected by centrifugation at 4,000× g for 10 min. Cells were washed, resuspended in PBS, and then inactivated at 65°C for 90 min. Each heat-inactivated whole bacterial sample was quantified by total protein concentration using BCA reagent (Thermo Scientific, Rockford, IL). The heat-inactivated *Burkholderia* bacteria (100 µg each) [BP 8324, BM ATCC 23344, and BT ATCC 700388] were divided into 3 micro-tubes. Heat-killed bacteria (BP, BM, and BT) were prepared by boiling for 10 min. To prepare the sodium periodate-treated bacteria (BP, BM, and BT), the cells were incubated with 20 mM sodium periodate in 50 mM sodium acetate buffer, pH 4.5 for 1 h at room temp in the dark. Following a brief rinse with 50 mM sodium acetate, the preparation was then incubated with 50 mM sodium borohydride in PBS for 30 min at room temp. To prepare the proteinase K-treated bacteria (BP, BM, and BT), the cells were incubated in 1 mg of proteinase K (Invitrogen) in 1 ml in distilled water at 60°C for 2 h. After incubation, 1 mM phenylmethylsulfonyl fluoride (PMSF; Sigma, St. Louis, MO) was added for 10 min, and then bacteria were washed three times in PBS. Another set of bacteria was treated with 0.02% SDS to maintain intact bacteria cell morphology.

### ELISA

For screening of positive clones by transfection, antibody quantification, and relative binding analysis, 96-well plates were coated with heat-killed Burkholderia bacteria at 100 ng of protein/well in 50 mM Na-bicarbonate buffer (pH 9.6) and blocked with 5% nonfat dry milk in the same buffer. One hundred microliters of undiluted (or normalized) cMAb supernatant was added to each well, including 100 µl each of both the positive (cMAb CK3) and negative (normal human serum, Sigma) controls at 1∶10 dilutions in PBS containing 1% BSA in 1×PBS. All plates were incubated at 37°C for 1 h and rinsed three times with PBS containing 0.05% Tween 20. Peroxidase-conjugated goat anti-human immunoglobulins (IgG+IgA+IgM; KPL, Gaithersburg, MD) at a 1∶2000 dilution in 1% BSA in 1×PBS were added and incubated at 37°C for 1 h. Substrate, ABTS system (KPL) was added and incubated at room temperature for 30 min. The optical density at 405 nm (OD_405_) and 492 nm was measured in an enzyme-linked immunosorbent assay (ELISA) plate reader (Molecular Device, Sunnyvale, CA). Each assay was repeated more than twice. The cutoff OD_405–492_ value for positive reaction was the mean OD_405–492_+3 standard deviations of negative control supernatant. Finally, all positive cells identified as positive clones by ELISA test were subcultured into six-well culture plates. When cells were confluent, these clones were frozen in 90% FBS and 10% dimethyl sulfoxide at −85°C. The wells identified as strong positive clones by primary screening were subcloned three times via a cloning cylinder or limiting dilution technique.

To quantify cMAb production levels, the capture antibody (Jackson ImmunoResearch Laboratories, West Grove, PA) against one human subclass immunoglobulin gamma1 Fc was coated (1 µg/well in 100 µl of 50 mM sodium carbonate buffer, pH 9.6) on 96-well ELISA plates. The plates were incubated with blocking solution (50 mM Tris buffer, pH 8.0 containing 0.14 M NaCl and 1% BSA) for 1 h. Hundred microliters of diluted samples or human immunoglobulin gamma 1 subclass standards (Sigma) were incubated in individual wells for 1 hr. The plates were then incubated with the HRP-conjugated specific IgG1-Fc detection antibodies (Jackson ImmunoResearch Laboratories) for 1 h. The plate was washed 5 times with PBS, and then colorized by substrate ABTS (KPL). The absorbance at 405 nm was measured on an ELISA plate reader (Molecular Device).

To analyze the binding properties of 3 cMAbs, exponentially growing bacteria (BP, BM, and BT) were inactivated by incubating in a 60°C water bath for 90 min. The bacteria were harvested by centrifugation and washed 6 times with PBS. Heat-killed Burkholderia bacteria (100 ng of protein/well) in sodium carbonate buffer were coated on ELISA plates and blocked with 5% nonfat dry milk. One hundred microliters of undiluted (or normalized antibody) supernatant including controls were added in the first row and serially diluted in a 96-well plate. The plates (100 µl in each well) were then incubated with the HRP-conjugated goat anti-human IgG (KPL) for 1 h. The plate was washed with 4 times PBS, and then colorized by substrate ABTS. The binding activities of 3 cMAbs were examined by their titration curve.

### SDS-PAGE and Western blot analysis

Ten micrograms of proteins of heat-killed, sodium periodate-treated, proteinase K-treated and outer membrane protein (OMP) fraction of each *Burkholderia* bacteria (BP, BM, and BT), were dissolved in sample buffer (5% 2-mercaptoethanol, 10% glycerol, 2% SDS, and 0.08% bromophenol blue in 62.5 mM Tris buffer [pH 6.8]). Samples, including 10 µl of diluted (1∶20) broad range molecular weight standards (Bio-Rad), were boiled at 100°C for 5 min. SDS-PAGE and Western blotting were performed as described elsewhere [29]. In brief, protein blots were immersed in blocking buffer (5% nonfat dry milk in 2×PBS) at 4°C overnight and incubated with culture supernatants (1∶10 dilution in blocking buffer) of cMAbs (CK1, CK2, and CK3) at room temperature for 2 h. After three rinses with TNTT buffer [50 mM Tris-HCl, 150 mM NaCl, 0.02% Tween 20, and 0.01% thimerosal (Sigma), pH 7.4], the blots were incubated at room temperature for 2 h with peroxidase-conjugated goat anti-human immunoglobulins (IgG+IgA+IgM) (KPL) at a 1∶2,000 dilution in blocking buffer. The blots were washed three times with TNTT buffer for 5 min each. The peroxidase-positive bands were detected by immersing the blots in a developing solution 4CN substrate (KPL) for 5 min. The enzyme reaction was terminated by washing the blots in 0.1 M H_2_SO_4_.

## Results

### Establishment of mammalian expression systems

The three MAbs BP7 10B11, BP7 2C6, and BP1 7F7 were selected for chimeric engineering [30], [31] based on our previous studies on binding specificity, relative antibody ranking, in vitro opsonic activity, and mouse protection against BP and/or BM. To obtain high level expression constructs, we modified the bicistronic pIRES vector by introducing respective DNAs of chimeric heavy (light) chains (H1∼H3; L1∼L3) (Table 1) into the multiple cloning site (MCS)-A. Into the MCS-B site, separated from the MCS-A by an IRES sequence, the DHFR gene was inserted to generate the pIRES-DHFR vector (Fig. 1A). Recently, a new development in cell transfection technology through the DHFR amplification allows the highly efficient transfection of mammalian cells relevant for production of neutralizing cMAbs against BP and/or BM infection (Fig. 1B). The key challenges in the development of stable cell lines able to produce cMAbs are summarized in the schematic chart (Fig. 1C).

To introduce the chimeric heavy or light chains into the MCS-A in the pIRES-DHFR plasmid, each chimeric fragment (mVH1∼3+HcG1 and mVL1∼3+HcK) of the 3 cMAbs CK1, CK2, and CK3 (Table 1) was assembled by the overlapping polymerase chain reaction (PCR) technique [32], [33] and sequenced. Multiple sequences of each chimeric chain were aligned by FRs, CDRs, junction between mouse variable gene and human constant genes (HcG1 or HcK) indicated on the basis of charge difference of amino acids in each chain (Fig. 2). The exact junctions of FR4 and human constant regions in each chimeric chain were pinpointed. Amino acid sequence homology data showed 89.6% sequence identity and 93.6% sequence similarity in these 3 V_H_ genes originating from the three different hybridoma clones and 82.9% sequence identity and 89.9% sequence similarity in the three Vκ chains.

To establish a series of technology for production of stable cMAbs and to evaluate which type of expression vector gives us a maximum performance for cMAb production, four major different expression systems with a DHFR amplification marker were generated by re-engineering the pIRES-DHFR vector. Overall, 6 vectors (4 single-gene vectors pIRES-H(L)-DHFR and p2A-H(L)-DHFR, and 2 double-gene vectors (pH-Fu-2A-L-DHFR and pIRES-L-H-DHFR)] (Fig. 3) were constructed to examine their performance. In brief, the bicistronic pIRES-H(L)-DHFR system allows translation of two consecutive open reading frames from the same messenger RNA due to the IRES element between the two transgenes. Recently, other investigators have reported that a gene transcribed upstream of the IRES is expressed strongly whereas a gene placed downstream is expressed at lower levels [34]. Since the cMAb production using the pIRES-H(L)-DHFR system might have some limitations- their size and variability in expression of two genes (cMAb and DHFR), we introduced the respective DNAs of chimeric heavy (light) chains into only MCS-A, not MCS-B of the pIRES-DHFR vector (Fig. 3A). Since the self-processing, FMDV-2A sequence (APVKQTLNFDLLKLAGDVESNPGP: 72 bp) is small sized compared to the internal promoter sequence with a CMV promoter enhancer (740 bp) or IRES sequence (581 bp), the 2A mediated co-expression strategy [35] using the p2A-H(L)-DHFR vectors may be significantly improved over the approach using the IRES system (Fig. 3B). The main difference between the single gene vectors and the double-gene vectors (pH-Fu-2A-L-DHFR and pIRES-L-H-DHFR) is that the amplifiable DHFR gene of both double-gene vectors is contained in the neomycin cassette, not in the MCS-B. In the pH-Fu2A-L-DHFR plasmid, the furin cleavage sequence (RAKR) [27] between the chimeric heavy chain and the 2A sequence was added to eliminate potential any adverse effects that might be caused by having a 2A peptide residue on our cMAbs due to incomplete cleavage of 2A-mediated heavy chain, which might lead to a significant decrease of cMAb production levels.

Another double-gene vector system (pIRES-L-H-DHFR) contains IRES sequences between the junctions of light (at MCS-A) and heavy (at MCS-B) chains (Fig. 3C). The unique restriction sites between the junctions in all eight vectors, sequences for synthetic leader and Kozak (except for the pH-Fu2A-L-DHFR vector) were also included to facilitate the vector engineering for future use of any drug candidates such as recombinant vaccines or proteins. Although these vector systems offer many unique qualities and advantages, the three single-gene vector systems were basically used for cotransfection of the two vectors (heavy and light chain) into the mammalian host cells, while the two double-gene vectors were used for single gene transfer.

### Optimization of transfection and selection

In a transient expression condition, we have predetermined the optimum transfection conditions [DNA concentration (total 10 µg/10-cm culture dish), molar ratios (H∶L = 1∶1 or 1∶2) (data not shown), use of lipofectamine (LF)-LTX reagent, and CHO-K1 cells among eight different host cells tested]. To further develop into the stable production of 3 cMAbs, not transient production, the CHO double deletion mutant (dhfr^−^/dhfr^−^) CHO-DG44 cells were transfected using a LF-LTX containing the PLUS reagent at a high cell density (more than 90% confluent in a 10-cm culture dish). The best results in transfection efficiency (60–90%) and positive selection through a step-wise DHFR amplification process were obtained, suggesting that the LF-LTX reagent is less toxic to the cells than the LF-2000 reagent, and the use of highly confluent cells in the culture dish had better chance to recover 16 h post-transfection due to close contact of adjacent cells in a 10 cm-culture dish. After more than 30 positive clones were identified by ELISA and cultured in a DHFR selective medium containing 0.005 µM MTX, the top 5 highest producers were further selected for the next amplification step after regaining their polygonal morphology (2–3 weeks). Although the concentration of MTX in our initial plan was expected to increase from 0.005 µM to 80 µM (4 times increase in each step), we found that the longer-incubation periods at high dose of MTX levels (>64 µM) resulted in decreased cMAb production level due to slowed growth rates. Therefore, these three cMAb producing stable clones were amplified only until 2 µM MTX levels for increase of cell growth rate and reduction of cell cytotoxicity in the final products.

### Binding activity of 3 cMAbs

**Figure 4:**
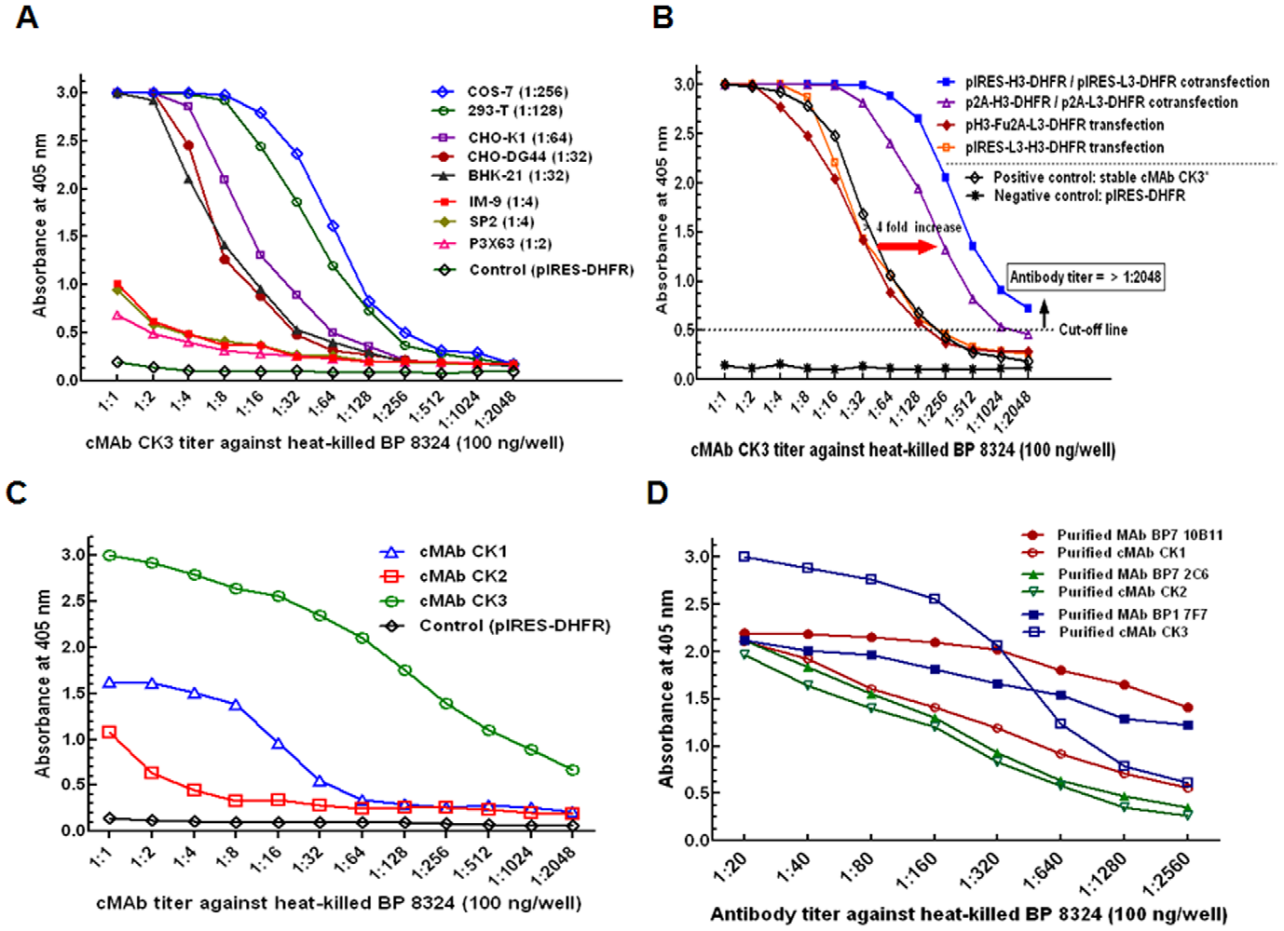
Binding activity of cMAbs produced by mammalian cells with different vector systems. (A) Selection of suitable host cells for transient production of cMAbs. The cMAb CK3 was transiently produced by 8 different mammalian host cells (∼5×10^6^ cells each/10 cm dish) for 5 days post-transfection using a LF-2000 reagent. The undiluted supernatants including a control (supernatant from CHO-K1 cells transfected by only pIRES-DHFR vector without chimeric chains), were used for cMAb CK3 titration. The number in the parenthesis mark indicates the cMAb CK3 titer. (B) Performance of the re-engineered vector systems for cMAb CK3 production was evaluated by CHO-K1 cells transfected using a LF-LTX reagent. Five days post-transfection of the vectors (10 µg) into the cells (∼1×10^6^ cells/10 cm dish), the undiluted supernatants were used for cMAb CK3 titration, including controls (stable cMAb CK3* at 2 µM MTX: pIRES-H3-DHFR/pIRES-L3-DHFR and culture supernatant by only pIRES-DHFR vector). (C) Binding property of 3 cMAbs produced by CHO-DG44 cells without DHFR amplification. For ELISA, the pIRES-H(L)-DHFR vectors containing chimeric H-chain (H1, H2, and H3) or L-chain (L1, L2, and L3) were cotransfected into CHO-DG44 cells (∼10^6^ cells/10 cm dish) using a LF-2000 reagent and the master seed cells were incubated for 3 days to get sufficient amounts of cMAbs before the DHFR-amplification process. (D) Comparison of binding property of stable cMAbs with the original mouse MAbs. Affinity-purified antibodies were adjusted to the same concentration starting with 5 µg/ml.

To examine the most suitable host cells for cMAb production by the pIRES-H(L)-DHFR vector systems, the cMAbs were transiently produced by 8 different mammalian host cells using a LF-2000 reagent. Five days after the transfection, the antibody production levels were measured by the human IgG1 ELISA. The MAb CK3 produced by COS-7 and 293T cells showed strong antibody titers (1∶256; 1∶128) (Fig. 4A) and higher production levels (∼400 ng/ml) than those levels produced by 6 different cells tested (Table 2).

Next, to test the re-engineered vector performance, the MAb CK3 was transiently produced by CHO-K1 cells using the LF-LTX reagent. The cMAb CK3 by the bicistronic single gene vectors [pIRES-H(L)-DHFR and p2A-H(L)-DHFR] had strong binding properties compared to stable cMAb CK3 at 2 µM MTX levels and transient cMAb CK3 by other 2 double gene vectors (Fig. 4B). The cMAb CK3 by CHO-K1 cells co-transfected by either the p2A-H(L)-DHFR or the pIRES-H(L)-DHFR vectors using a LF-LTX reagent showed a strong binding activity against BP (Fig. 4B) and reached high production levels (1,049 and 694 ng/ml, respectively) compared to two other systems (Table 3).

**Table 3:** Production of cMAb CK3 using different expression vectors with a DHFR gene^a^.

Expression system	cMAb CK3
	Concn^b^ (ng/ml)
pIRES-H3-DHFR/pIRES-L3-DHFR	694±14
p2A-H3-DHFR/p2A-L3-DHFR	1,049±21
pH3-Fu2A-L3-DHFR	48±1
pIRES-L3-H3-DHFR	42±1

^*a*^To evaluate the expression performance using 5 different vector systems, each construct was transfected into CHO-K1 cells (∼10^6^ cells/10 cm dish) using a LF-LTX reagent. The cells were incubated for 72 h.

^*b*^Culture supernatants were used to measure cMAb CK3 levels by the ELISA. The values are the means ± standard deviations of 3 independent experiments.

Third, to examine the binding property of 3 cMAbs (cMAbs CK1, CK2, and CK3) produced by transfectoma CHO-DG44 cells using the pIRES-H(L)-DHFR vector system, the undiluted 3-day culture supernatant without DHFR amplification was titrated by ELISA using the heat-killed BP antigen. The result indicated that the cMAb CK3 showed a strong binding activity to BP over cMAbs CK1 and CK2 (Fig. 4C). The master seed cells were further incubated for 14 days in DHFR selection medium [(α-MEM without added nucleosides and dialyzed fetal bovine serum (dFBS)] to isolate the highest producers using cloning cylinders before entering the amplification process by MTX.

We also compared the binding property of the affinity-purified cMAb CK3 with the original mouse MAb BP1 7F7 by adjusting to the same concentration starting with 5 µg/ml. The binding affinity between the MAb CK3 and the MAb BP1 7F7 was not significantly changed from the dilutions 1∶1 to 1∶160, thereafter the slope of the cMAb CK3 titration curve was slightly dropped compared to the binding curve of MAb BP1 7F7 (Fig. 4D). This slight loss of binding activity to the BP antigen may be due to the differences in antibody assembly and post-translational modification between two host cells (CHO-K1 and hybridoma) during the chimerization process from the original mouse hybridoma BP1 7F7.

### Production and purification of cMAb CK3 to examine cMAb quality

The cMAb production levels by 8 different host cells and 4 different constructs are summarized in Table 2 and 3. Overall, the use of bicistronic pIRES-H(L)-DHFR expression vectors, containing the gene of either chimeric heavy or light chain upstream of the DHFR amplifiable marker, was effective in obtaining high levels of the transient cMAb produced by using CHO-K1 cells. The production levels (1,049 ng/ml) of a bicistronic vector based on 2A sequence was ∼1.5 times greater than that (694 ng/ml) of an IRES based vector.

**Figure 5:**
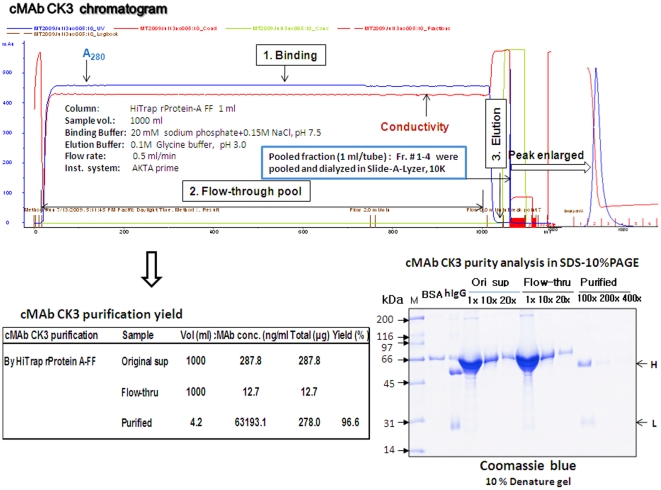
Affinity purification and purity analysis of cMAb CK3. For purification and purity analysis of cMAb, the pooled cMAb CK3 supernatant samples (∼1 L) were purified by the ÄKTAprime plus using an affinity chromatography column (HiTrap rProtein A-FF). The pooled cMAb fractions were dialyzed and purification yield were evaluated by the modified human IgG1 ELISA. The purity of cMAb CK3 was analyzed by the SDS-10% PAGE.

For purification and purity analysis of cMAb CK3, the pooled CK3 supernatant samples (∼1 L) were purified by the ÄKTAprime plus using an affinity chromatography column (HiTrap rProtein A-FF). The cMAb CK3 chromatogram consisted of the binding, flow-through, and elution (Fig. 5). The pooled cMAb fractions were dialyzed and purification yields were evaluated by the modified human IgG1 ELISA. The purity of cMAb CK3 was analyzed by SDS-10% polyacrylamide gel electrophoresis (PAGE). Analysis by ELISA and gel electrophoresis showed that cMAb CK3 was recovered at purity and final yield of 92–96% from the HiTrap rProtein A-FF column. The ÄKTAprime was operated by slow flow-rate (0.25–0.5 ml/min) and pH of binding buffer (20 mM sodium phosphate containing 150 mM NaCl, pH 7.5) for successful purification of cMAb CK3. Since the purity of cMAb obtained from HiTrap rProtein A-FF column was high, the ability to withstand high column pressures and nearly 95% recovery of the original supernatant antibody made a HiTrap rProtein A-FF column well suited as an initial capture step without a further polishing step. These results also indicated that cMAb CK3 was appropriately produced by CHO-DG44 cells through our expression systems and were heterodimeric form with a right size Fc tail necessary for binding to protein A column.

### Expression analysis of cMAb CK3

**Figure 6:**
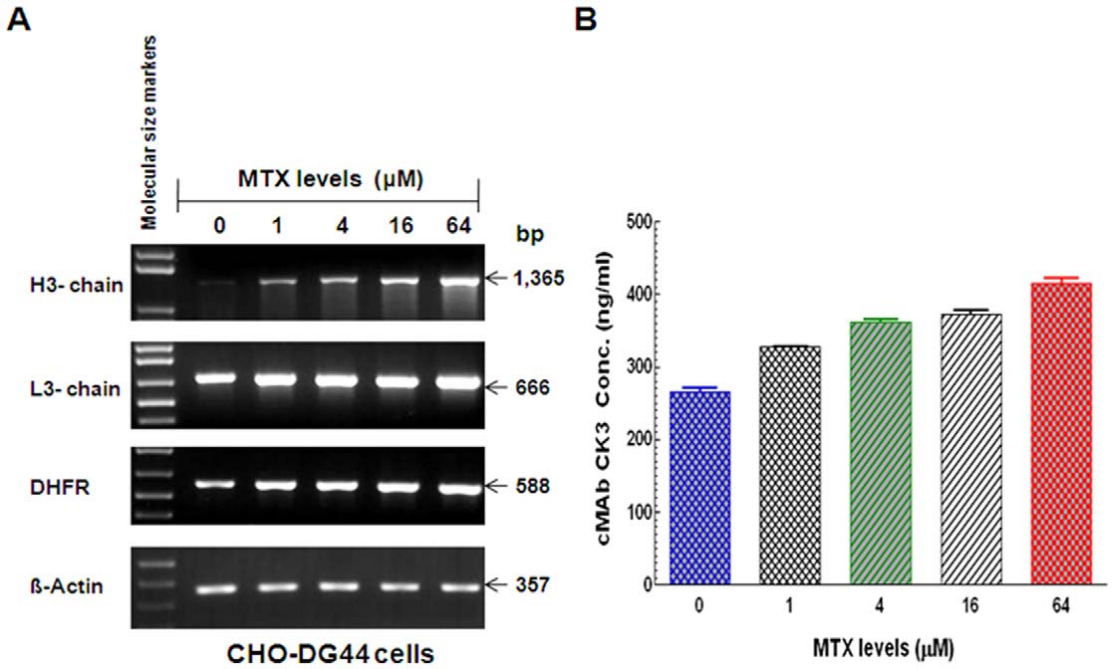
Dose-dependent upregulation of mRNAs (chimeric H3- and L3-chains, and DHFR) and cMAb CK3 production levels by stable CHO-DG 44 CK3 cells at different MTX levels. (A) mRNA (H3, L3, and DHFR genes) expression in the stable CHO-DG44 CK3 cells at the 4-fold increase of MTX levels (0, 1, 4, 16, and 64 µM) for 7 days. DNA size markers (1 Kb plus fragments) are shown at the left. The data presented are representative of 3 independent experiments that gave similar results. (B) MTX dose-dependent cMAb CK3 production by the stable CHO-DG44 at different MTX levels. The amounts of cMAb from CHO-DG44 cells (5×10^6^ cells) were measured by the modified human IgG1 ELISA.

Since we hypothesized that the amplifiable DHFR gene in the expression vector contributes to the increased amount of antibody gene expression, leading to the high-level production of stable cMAb, the effect of DHFR amplification on mRNA expression and cMAb CK3 production levels was examined by RT-PCR and ELISA. The results showed the dose-dependent upregulation of mRNAs (chimeric H3- and L3-chains, and DHFR) (Fig. 6A) and cMAb CK3 production levels (starting above 400 ng/ml without DHFR amplification) (Fig. 6B) at a 4-fold increase of MTX levels (0, 1, 4, 16, and 64 µM) for 7 days. In the stable CHO-DG44 cells, both the cMAb CK3 mRNA expression and production levels were increased in a MTX dose-dependent manner suggesting that the DHFR system would be useful for overproduction of therapeutic MAbs [36].

### Reactive antigen analysis by three cMAbs

**Figure 7:**
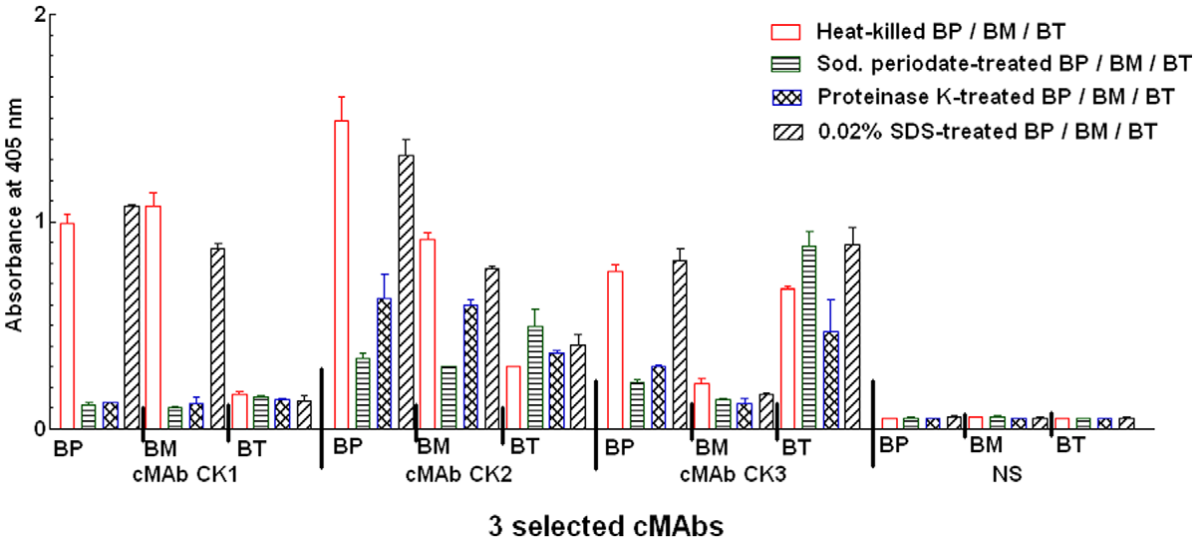
Reactive antigen analysis of 3 cMAbs using the treated-Burkholderia bacteria antigens. Three Burkholderia (BP 8324, BM ATCC 23344, and BT ATCC 700388) species were used to test the binding specificities of the anti-Burkholderia (cMAbs CK1, CK2, and CK3), and the antigen reactivities of the cMAbs to bacterial cell components. The cMAbs and normal human serum (NS: a negative control) were adjusted to the same concentration (5 µg/ml).

**Figure 8:**
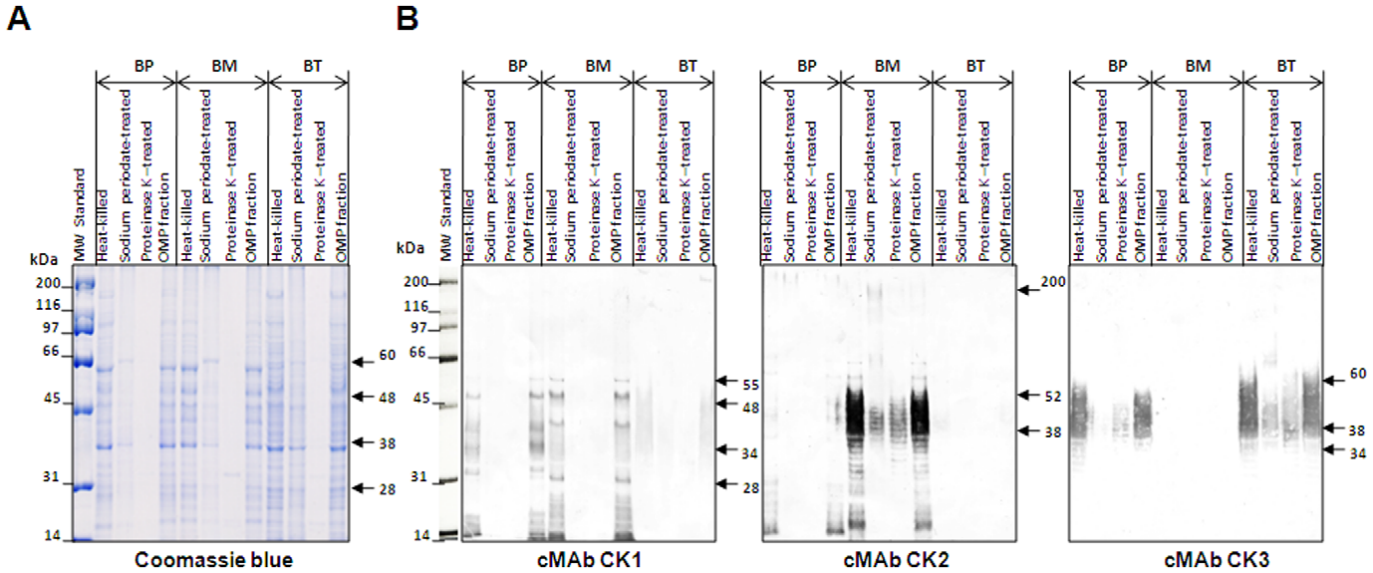
Western immunoblot analysis of BP, BM, and BT with 3 cMAbs. (A) Proteins were separated on an SDS-10% polyacrylamide gel and stained with Coomassie blue. (B) For Western blot analysis, 3 cMAb culture supernatants (cMAbs CK1, CK2, and CK3) were used at 1∶5 dilutions. Molecular weight (MW) standards were from Bio-Rad.

Three Burkholderia (BP 8324, BM ATCC 23344, and *B. thailandensis* (BT) ATCC 700388) species were used to test their binding specificities to 3 cMAbs (cMAbs CK1, CK2, and CK3), and the antigen reactivities to their bacterial cell components by ELISA and Western blot analysis using treated whole bacterial antigens [heat, sodium periodate [37] (to detect cMAb specific for carbohydrate epitopes using a periodate oxidation), proteinase K, 0.02% sodium dodecyl sulfate (SDS), and OMP fractions. ELISA data (Fig. 7) and Western blot results (Fig. 8) showed that the cMAb CK1 reacted with glycoproteins (22–28, 38, 48, 55 kDa in BP and BM). The cMAb CK2 recognized capsular polysaccharide antigens with molecular sizes of 37 to 51 kDa, and 200 kDa in BM. The cMAb CK2 was slightly reactive to 14–28 kDa and 200 kDa protein in BP. The cMAb CK3 reacted with typical LPS-like antigens (34, 38–52 kDa in BP or 34, 38–60 kDa in BT) (Fig. 7B, Table 4). Since mild treatment of the bacteria cells with 0.02% SDS could maintain bacterial membrane rigidity without cell disruption, the antibody reactivity to these epitopes had no significant differences compared to the heat-killed Burkholderia (Fig. 7). Western blot results with the OMP fraction of the 3 Burkholderia species were consistent with results of whole Burkholderia cell antigens (Fig. 8).

**Table 4:** Characteristics of 3 cMAbs against BP, BM, and BT.

cMAb ID	Antigen(s) (kDa) recognized in BP, BM, and BT^a^			Localization^b^
	BP	BM	BT	
cMAb CK1	32, **34**, **38**, **48**	22∼**28**, **38**, **48**, 55	NR^c^	Surface-Glycoprotein
cMAb CK2	14∼28, 200	**38∼52**, 200	NR	Surface-Capsule
cMAb CK3	34, **38∼52**	NR	34, **38∼60**	Surface-LPS

^*a*^Major antigens of the 3 Burkholderia species are shown in boldface type.

^*b*^Dependent on ELISA analysis and Western blot results by treatments (heat, sodium periodate, and proteinase K) and OMPs of 3 Burkholderia species.

^*c*^NR: Not reactive by WB, but weakly reactive by ELISA.

## Discussion

In this study, we developed a series of technology for production of stable cMAbs against BP and/or BM by CHO-DG44 cells using the DHFR expression systems. Overall, we have generated six mammalian expression vectors, incorporating IRES and FMDV-2A sequences that efficiently mediated the (co)expression of two transgenes in multiple mammalian cell types. These vectors are useful for various cMAb productions in the laboratory. In our present study, the utilization of either bicistronic pIRES-H(L)-DHFR or p2A-H(L)-DHFR vectors has been shown as the most effective method in obtaining stable cell lines that express high levels of the cMAbs. Therefore, two systems utilizing single gene vectors might be more relevant than other systems for maintaining intact binding properites after chimerization and higher level production of cMAbs by mammalian host cells.

Recent progresses in antibody engineering, cell transfection technology, and expression systems containing selective markers allow highly efficient transfection of mammalian cells for the fast production of neutralizing cMAbs [31], [38] The efficient transfection of cells not only facilitates stable cell clone generation, but also allows true preclinical development of cMAb production in transiently transfected cells for small-scale production. Using the CHO double deletion mutant (dhfr^−^/dhfr^−^) CHO-DG44 cells, we were able to amplify the chimeric antibody gene associated with the amplifiable DHFR gene by systematic increases of MTX in medium without added nucleosides. For the development of therapeutic cMAbs, it is critical to obtain a thoroughly guaranteed stable clone derived from a single clone producing stable cMAbs. Using the bicistronic pIRES-H(L)-DHFR vector system, approximately six months is usually required due to selection/amplification and adaptation to serum-free conditions. By using cloning cylinders and applying a less toxic lipofectamine (LF-LTX) reagent to the highly confluent CHO-DG44 cells (∼90%), we significantly reduced the time and increased the probability of finding the positive clone. Although tedious time is still required for these procedures, the end-results obtained through the selection/amplification processes allow us to use stable cMAbs.

Considering the production levels and binding properties of cMAbs produced by transient transfection using 8 different mammalian host cells, the cell lines (CHO-K1, COS-7, 293T, and BHK-21) were more productive than other mammalian cells tested. However, for large-scale production, the CHO-DG44 cells are only available in obtaining stable clones due to the property of deficient DHFR genes within host chromosomes. Thus, we can continuously amplify the target antibody gene up to certain MTX levels without causing any cell damage. When producing therapeutic cMAb, the CHO-DG44 cell line makes an ideal cell factory since their cMAbs largely correspond to genuine human MAbs with similar patterns of post-translational modification and glycosylation [38]. The availability of commercial media of high quality for cell culture is an additional factor that would support large-scale production of cMAbs in this cell line.

Recent studies of the role of *Burkholderia* outer surface antigens in mouse protection by using MAbs post-nasal or lethal challenges of pathogenic *Burkholderia* species indicate that the Burkholderia surface antigens (OMPs, LPS, and capsular polysaccharide) are potential protective antigenic targets [39]–[43]. Our ELISA data and Western blot analysis, using the treated antigens (heat, sodium periodate, proteinase K, and 0.02% SDS) and OMPs antigens of 3 Burkholderia species, revealed each cMAb's reactivity to bacterial cell components and immunodominant antigens with different molecular size from 21 kDa to 65 kDa. Briefly, due to different antigen reactivities (cMAb CK1: surface glycoproteins of BP and BM; cMAb CK2: capsular polysaccharide antigens of BP and BM; cMAb CK3: surface LPS and/or lipoproteins (LP) of BP and BT), these 3 cMAbs would be useful for neutralizing BP and/or BM infection and studying the role of the major surface antigens in Burkholderia infection. Furthermore, since LP is a major component of the outer membrane of gram-negative bacteria, LP in culture supernatants of growing Burkholderia cells may induce pathologic changes associated with infections [44]. Our previous studies of in vitro neutralization and antibody ranking using the original mouse MAbs suggest that the surface epitopes (with little cross-reactivity to other Burkholderia bacteria) recognized by these 3 cMAbs are highly specific to target *Burkholderia* species. Thus, these cross-reactive antigens in 3 *Burkholderia* species may be useful for determining potential vaccine targets against Burkholderia infection.
